# Supplementary material for: Reversal of cell, circuit and seizure phenotypes in a mouse model of DNM1 epileptic encephalopathy
Source: Nat Commun. 2023 Aug 30;14:5285. doi: 10.1038/s41467-023-41035-w (PMC10468497; doi:10.1038/s41467-023-41035-w)
Supplement: Supplementary file 1 — Supplementary Information [file 41467_2023_41035_MOESM1_ESM.pdf]

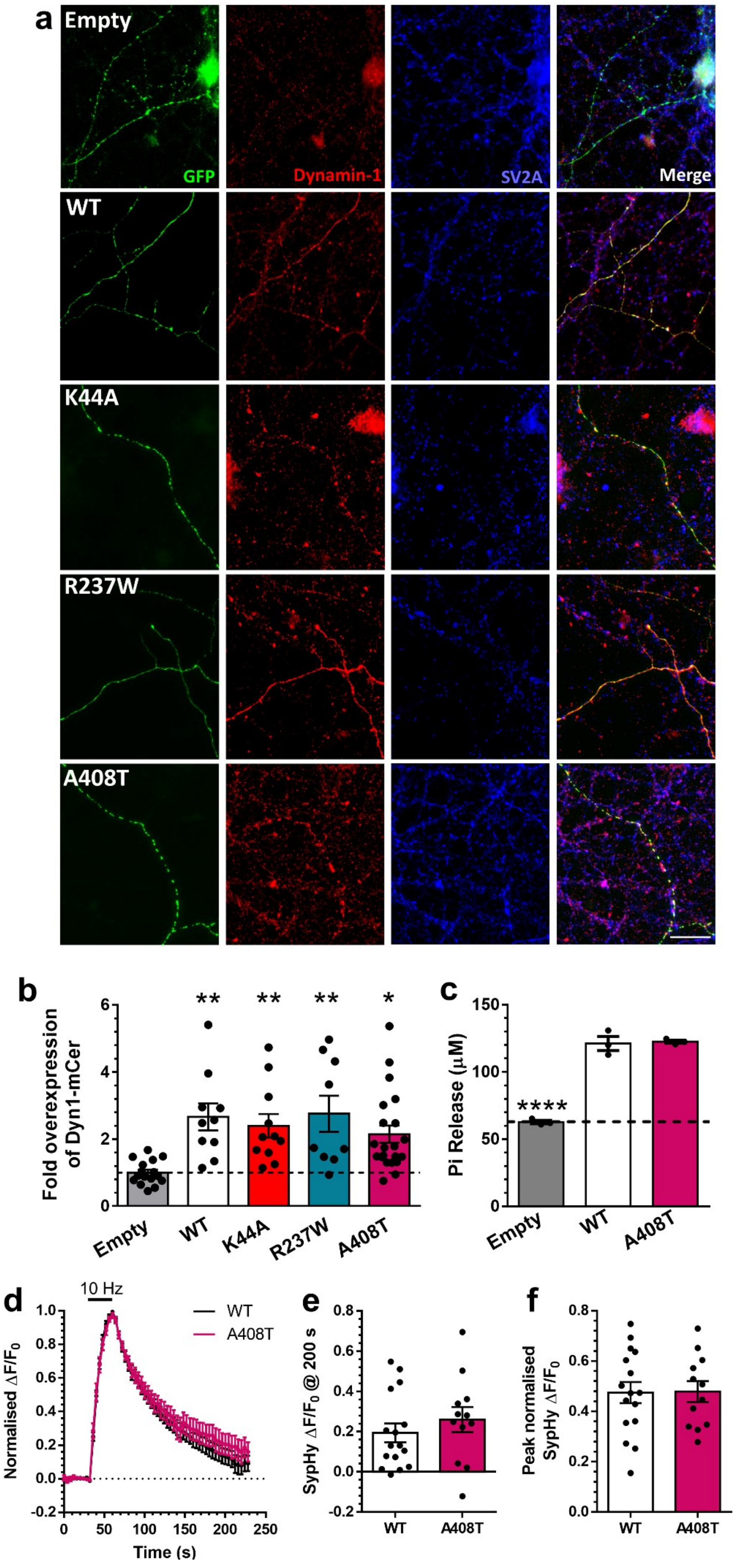
a
Empty
GFP
Dynamin-1
SV2A
Merge
WT
K44A
R237W
A408T
b
Fold overexpression of Dyn1-mCer
** ** ** *
Empty WT K44A R237W A408T
c
Pi Release (μM)
****
Empty WT A408T
d
10 Hz
WT
A408T
Normalised ΔF/F0
Time (s)
e
SypHy ΔF/F0 @ 200 s
WT A408T
f
Peak normalised SypHy ΔF/F0
WT A408T

Supplementary Figure 1 – *Expression levels of $Dyn1_{WT}$-mCer plasmids and lack of effect of A408T dynamin-1 mutant.* (**a,b**) Primary cultures of hippocampal neurons were transfected with synaptophysin-pHluorin (sypHy) and either mCer (Empty), $Dyn1_{WT}$-mCer, $Dyn1_{K44A}$-mCer, $Dyn1_{R237W}$-mCer or $Dyn1_{A408T}$-mCer between 11-13 DIV. At 13-15 DIV, cultures were fixed and immunostained for the presence of mCer (GFP), dynamin-1 and SV2A. (**a**) Representative images, scale bar = 20 μm. (**b**) Average fold over-expression of Dyn1-mCer plasmids, normalised to endogenous dynamin-1 levels ± SEM. One-way ANOVA, n=16 Empty, n=10 $Dyn1_{WT}$-mCer, n=11 $Dyn1_{K44A}$-mCer, n=9 $Dyn1_{R237W}$-mCer, All against Empty ** p=0.0015 $Dyn1_{WT}$-mCer, ** p=0.0065 $Dyn1_{K44A}$-mCer, ** p=0.0012 $Dyn1_{R237W}$-mCer, * p=0.116 $Dyn1_{A408T}$-mCer. (**c**) HEK293T cells were transfected with either mCer (Empty), $Dyn1_{WT}$-mCer or $Dyn1_{A408T}$-mCer. After 48 h the cells were lysed and mCer was immunoprecipitated. The GTPase activity of the immunoprecipitate is displayed as released Pi ± SEM (one-way ANOVA, all n=3 separate experiments, ****p<0.0001 WT to Empty, p=0.9873 WT to A408T). (**d-f**) Primary cultures of hippocampal neurons were transfected with sypHy and either $Dyn1_{WT}$-mCer or $Dyn1_{A408T}$-mCer between 11-13 DIV. At 13-15 DIV, cultures were stimulated with a train of 300 action potentials (10 Hz). Cultures were pulsed with NH_4Cl imaging buffer 180 s after stimulation. (**d**) Average sypHy response ($\Delta F/F_0$ ± SEM) normalised to the stimulation peak. Bar indicates stimulation (n=16 $Dyn1_{WT}$-mCer, n=12 $Dyn1_{A408T}$-mCer). (**e**) The average level of sypHy fluorescence ($\Delta F/F_0$ ± SEM) at 200 s (Unpaired two-sided t test, n=16 $Dyn1_{WT}$-mCer, n=12 $Dyn1_{A408T}$-mCer, p=0.397). (**f**) The peak level of sypHy fluorescence ($\Delta F/F_0$ ± SEM) normalised to the NH_4Cl challenge (Unpaired two-sided t test, n=16 $Dyn1_{WT}$-mCer, n=12 $Dyn1_{A408T}$-mCer, p=0.948). Source data are provided as a Source Data file.

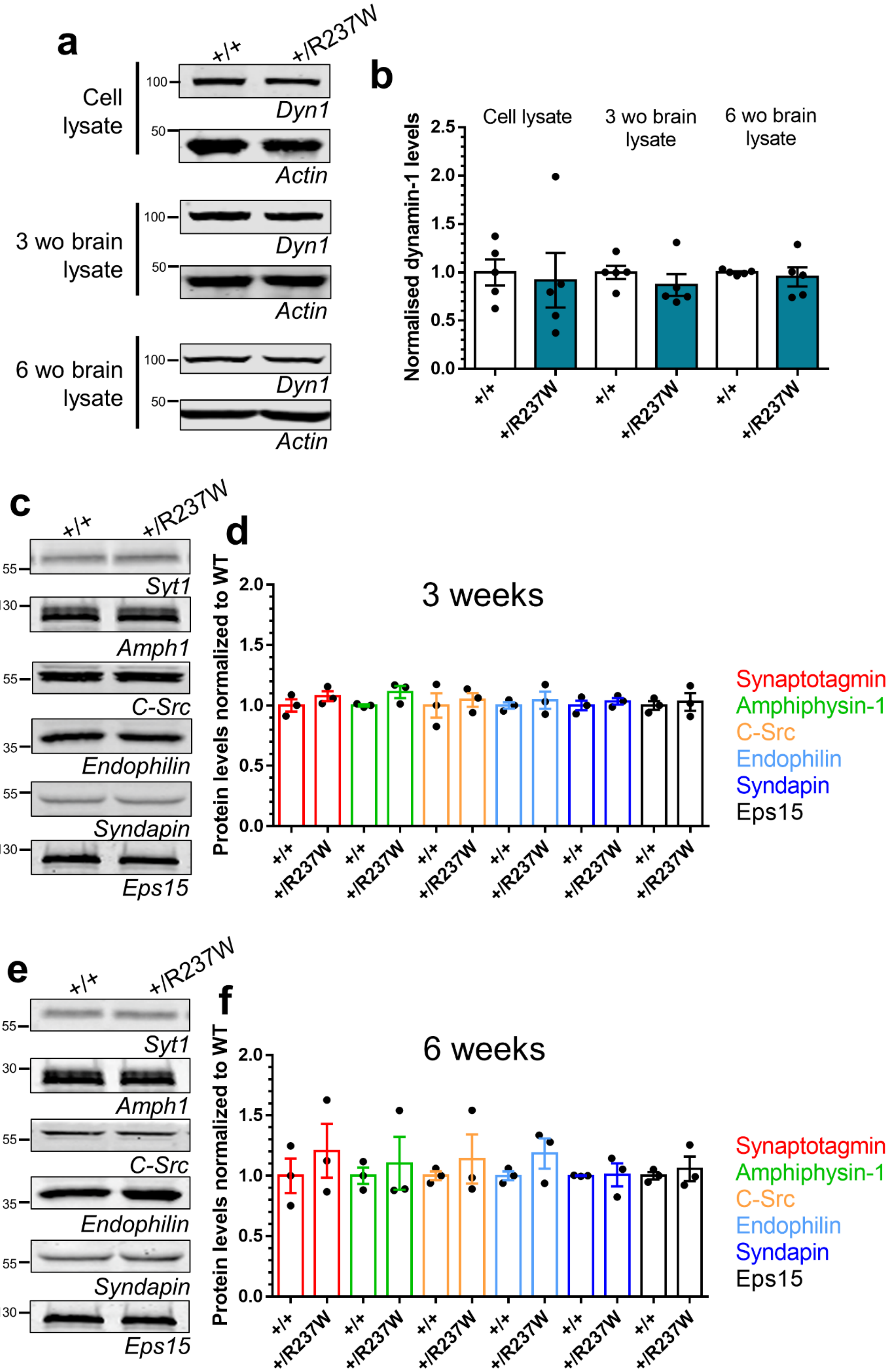
a
+/+
+/R237W
Cell lysate
3 wo brain lysate
6 wo brain lysate
100
50
Dyn1
Actin
b
Cell lysate
3 wo brain lysate
6 wo brain lysate
Normalised dynamin-1 levels
2.5
2.0
1.5
1.0
0.5
0.0
c
55
130
35
Syt1
Amph1
C-Src
Endophilin
Syndapin
Eps15
d
3 weeks
Protein levels normalized to WT
Synaptotagmin
Amphiphysin-1
C-Src
Endophilin
Syndapin
Eps15
e
f
6 weeks

Supplementary Figure 2 – *Dynamin-1 and other synaptic protein levels in Dnm1$^{+/R237W}$ mice.* Lysates were generated from either primary cultures of either *Dnm1*$^{+/+}$ or *Dnm1*$^{+/R237W}$ hippocampal neurons or from the brains of either 3 week or 6 week old *Dnm1*$^{+/+}$ or *Dnm1*$^{+/R237W}$ mice. (**a**) Representative blots for dynamin-1 levels in *Dnm1*$^{+/+}$ or *Dnm1*$^{+/R237W}$ lysates. (**b**) Average levels of dynamin-1 normalised to *Dnm1*$^{+/+}$ lysates ± SEM (Unpaired two-sided t test with Welch's correction, all n=5 samples, p=0.801 cell lysate, p=0.359 3 week, p=0.671 6 week). (**c,e**) Representative blots display levels of Synaptotagmin-1 (Syt1), Amphiphysin-1 (Amph1), C-src, Endophilin, Syndapin and Eps15 in either 3 week (**c**) or 6 week (**e**) lysates. (**d,f**) Quantification of protein levels normalised to *Dnm1*$^{+/+}$ ± SEM in either 3 week (**d**) or 6 week (**f**) lysates (n=3 independent brain lysate preparations for all, all ns, two-sided Mann-Whitney test). Source data are provided as a Source Data file.

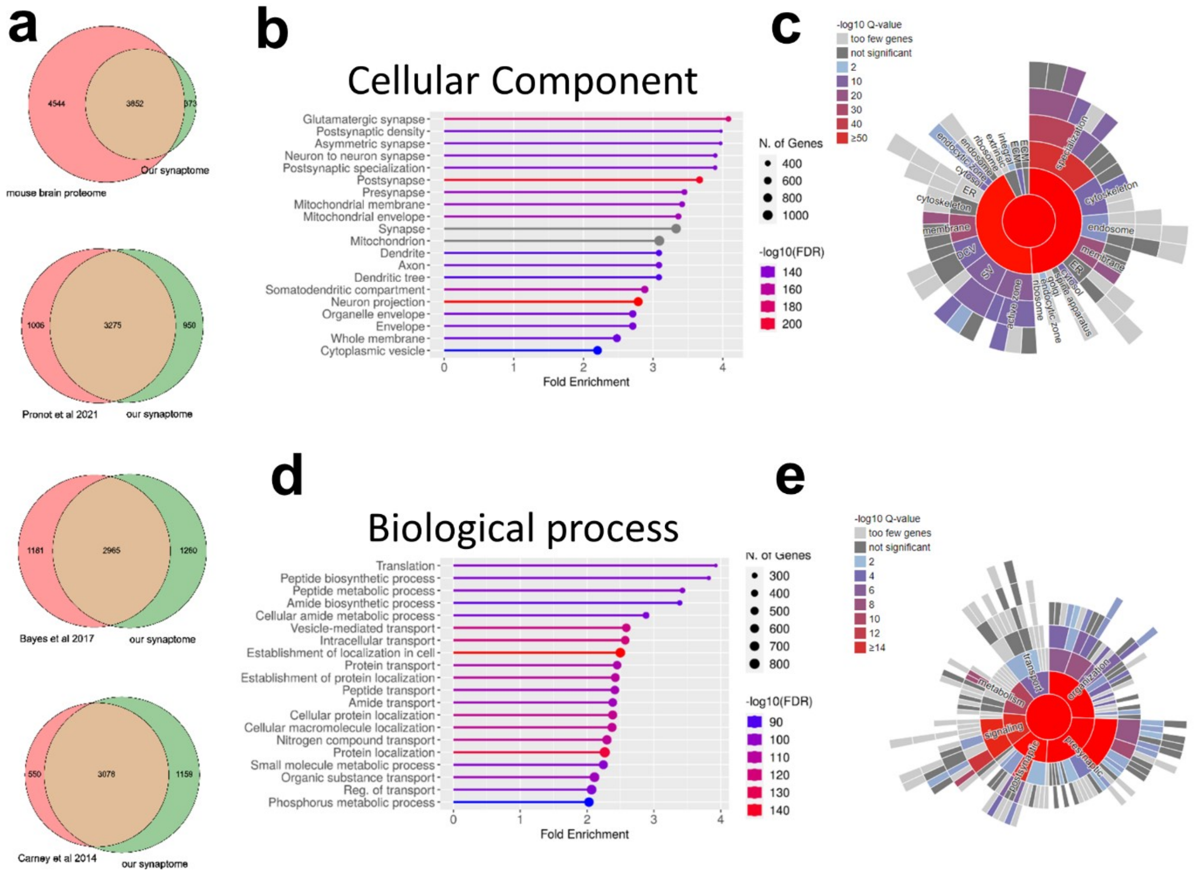

Supplementary Figure 3 – *Proteomic data.* (**a**) Venn diagrams showing overlap between the synaptome of this study and other published synaptosome proteomes. Terms enrichment analysis of 4237 synaptic proteins found by MS for GO Cellular Components (**b**) and Biological Process (**d**) using ShinyGO databases and for the Cellular Components (**c**) and Biological Pathways (**e**) using SynGO synaptic component curator tool. Source data are provided as a Source Data file.

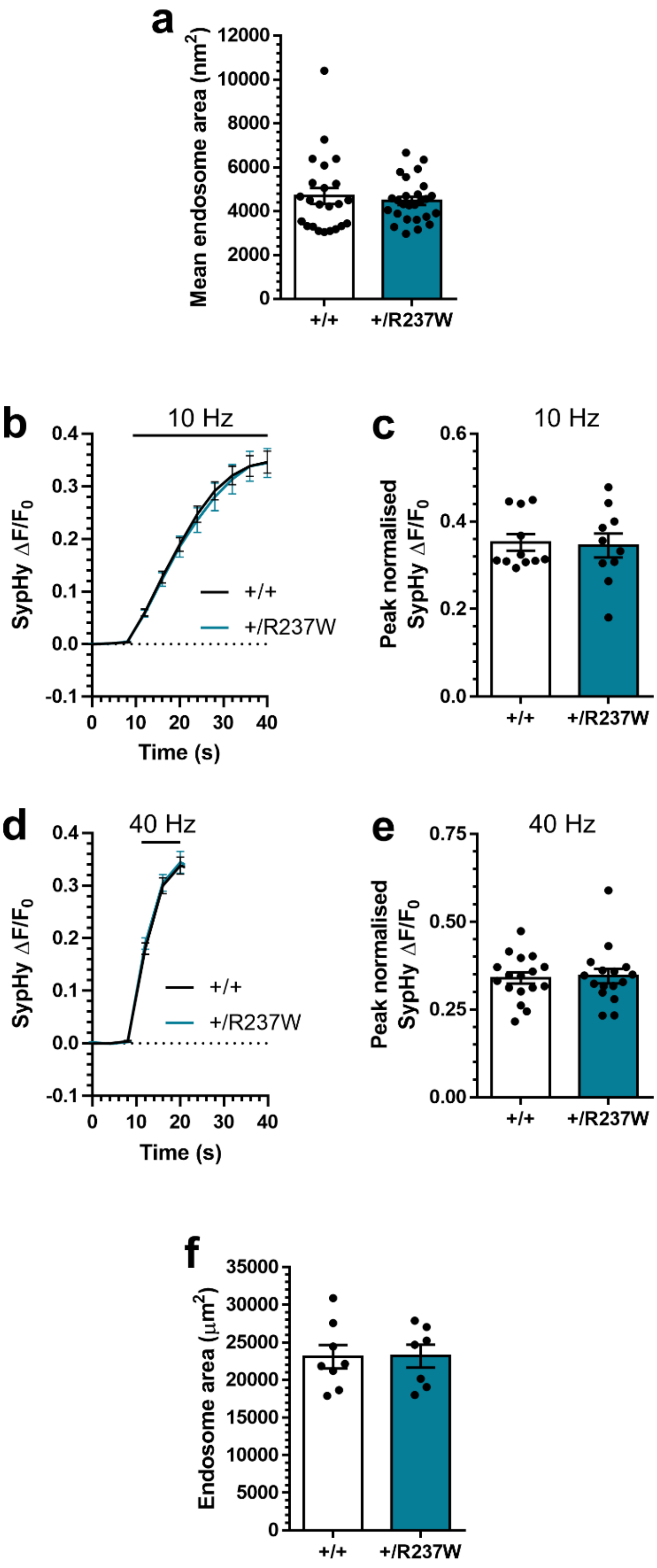

Supplementary Figure 4 – *SV pools and endosomes in $Dnm1^{+/R237W}$ mice.* (**a**) Brains from 2 month-old $Dnm1^{+/+}$ and $Dnm1^{+/R237W}$ mice were perfusion fixed and processed for electron microscopy. The size of presynaptic endosomes is displayed ± SEM (n=23 profiles $Dnm1^{+/+}$, n=26 $Dnm1^{+/R237W}$, p=0.891 two-sided Mann-Whitney test). (**b-e**) Primary cultures of hippocampal neurons prepared from either $Dnm1^{+/+}$ and $Dnm1^{+/R237W}$ embryos were transfected with synaptophysin-pHluorin (sypHy) between 7-9 DIV. At 13-15 DIV, cultures were stimulated with a train of either (**b,c**) 300 action potentials (10 Hz) or (**d,e**) 400 action potentials (40 Hz) in the presence of 1 µM bafilomycin-A1. Immediately after stimulation, cultures were pulsed with NH_4Cl imaging buffer. (**b,d**) Average sypHy response (ΔF/F_0 ± SEM) to either 10 Hz (**b**) or 40 Hz (**d**) stimulation, normalised to the NH_4Cl challenge peak (**b**, n=11 $Dnm1^{+/+}$, n=10 $Dnm1^{+/R237W}$, p=0.839; **d**, n=17 $Dnm1^{+/+}$, n=16 $Dnm1^{+/R237W}$). (**c,e**) Peak level of sypHy fluorescence (ΔF/F_0 ± SEM) normalised to the NH_4Cl challenge (**c** Unpaired two-sided t test, n=11 $Dnm1^{+/+}$, n=10 $Dnm1^{+/R237W}$, p=0.839; **e** Two-sided Mann-Whitney test, n=17 $Dnm1^{+/+}$, n=16 $Dnm1^{+/R237W}$, p=0.919). (**f**) $Dnm1^{+/+}$ and $Dnm1^{+/R237W}$ neurons were stimulated with a train of 400 action potentials (40 Hz) in the presence of 10 mg/ml HRP. Average number HRP-labelled endosome diameter is displayed ± SEM (Unpaired two-sided t test, n=8 $Dnm1^{+/+}$, n=7 $Dnm1^{+/R237W}$ p=0.974). Source data are provided as a Source Data file.

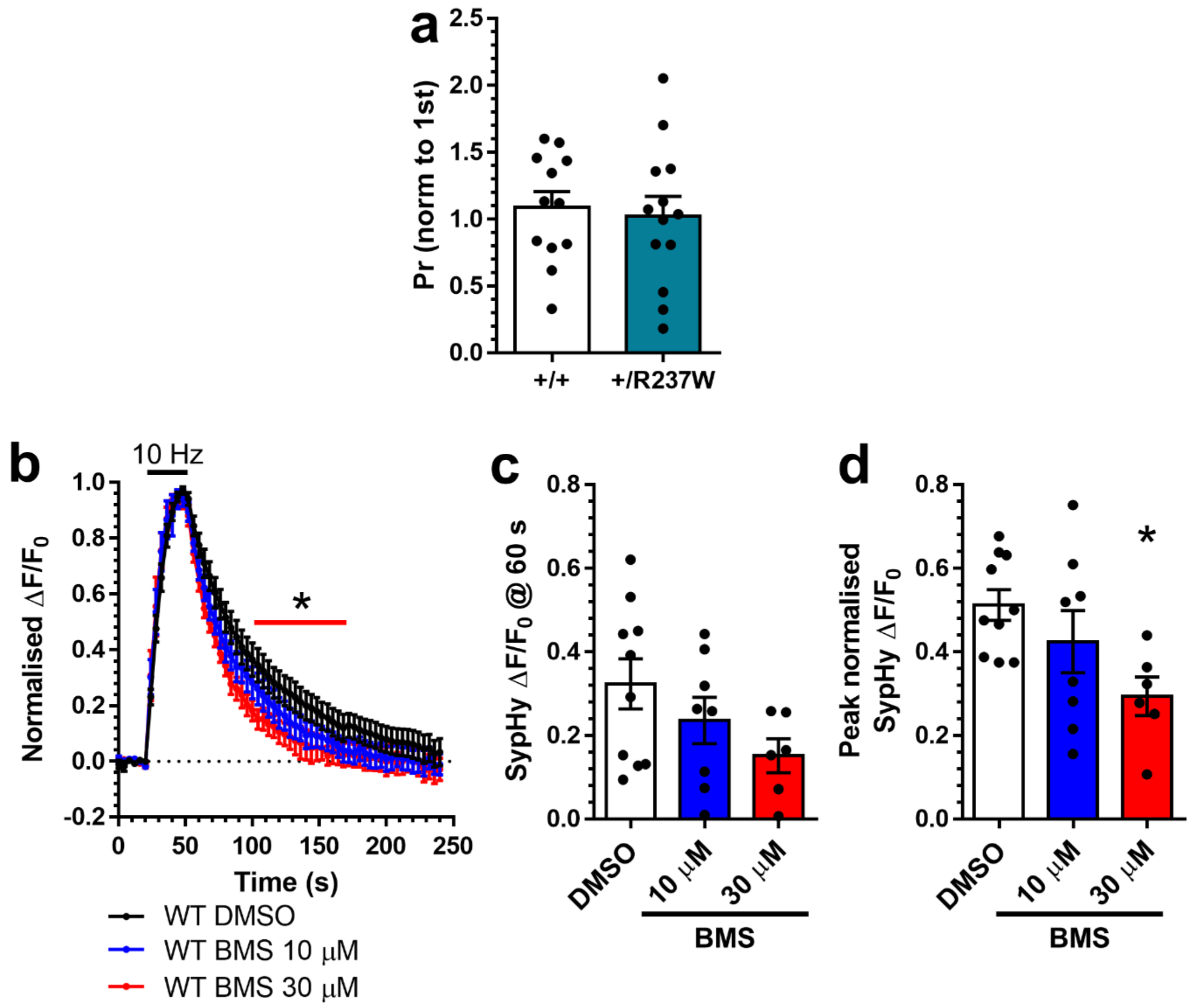

Supplementary Figure 5 – *BMS-204352 accelerates SV endocytosis.* (**a**) Neurotransmission at CA3/CA1 synapses was monitored using whole-cell patch clamp recording in acute hippocampal slices from *Dnm1*$^{+/+}$ and *Dnm1*$^{+/R237W}$ mice. Slices were stimulated with 600 APs (40 Hz, Figure 5**f,g** of main manuscript). Mean Pr was calculated by dividing the amplitude of the first evoked EPSC by the effective RRP size ± SEM (n=12 *Dnm1*$^{+/+}$, n=13 *Dnm1*$^{+/R237W}$, unpaired two-sided t test, p=0.739). (**b-d**) Primary cultures of hippocampal neurons prepared from *Dnm1*$^{+/+}$ embryos were transfected with synaptophysin-pHluorin (sypHy) and Dyn1$_{WT}$-mCer between 11-13 DIV. At 13-15 DIV, cultures were stimulated with a train of 300 action potentials (10 Hz) in the presence of either 10 μM or 30 μM BMS-204352 or a vehicle control (DMSO). Cultures were pulsed with NH_4Cl imaging buffer 180 s after stimulation. (**b**) Average sypHy response ($\Delta F/F_0$ ± SEM) normalised to the stimulation peak. Bar indicates period of stimulation. Two-way ANOVA *p< 0.05 (n=10 DMSO, n=8 10 μM, n=6 30 μM). (**c**) Average level of sypHy fluorescence ($\Delta F/F_0$ ± SEM) at 60 s (One-way ANOVA, n=10 DMSO, n=8 10 μM, n=6 30 μM, all ns). (**d**) Peak level of sypHy fluorescence ($\Delta F/F_0$ ± SEM) normalised to the NH_4Cl challenge (One-way ANOVA, n=10 DMSO, n=8 10 μM, n=6 30 μM, *p=0.0217 DMSO vs 30 μM). Source data are provided as a Source Data file.

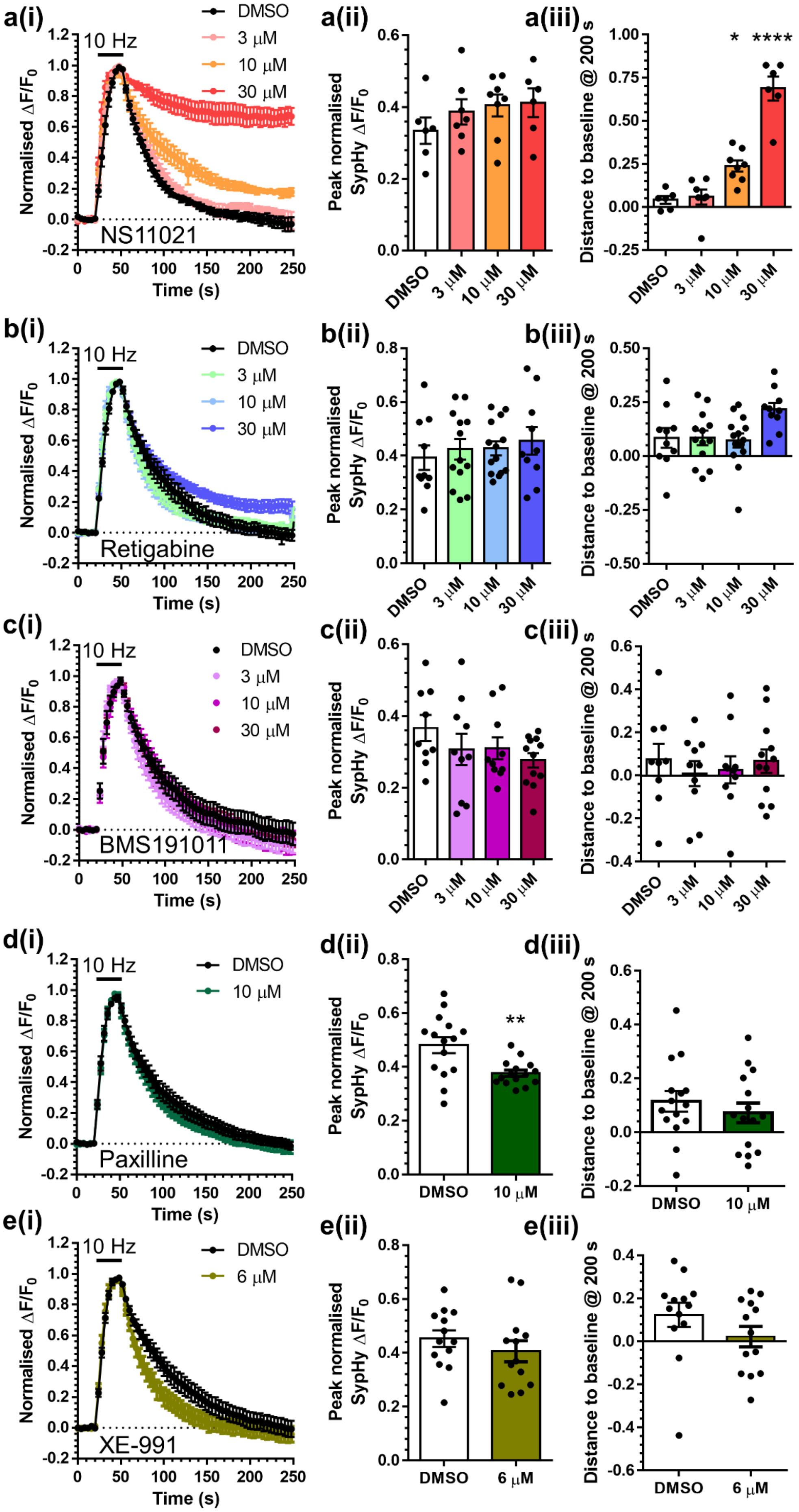
a(i)
a(ii)
a(iii)
10 Hz
DMSO
3 μM
10 μM
30 μM
NS11021
Normalised ΔF/F0
Time (s)
Peak normalised SypHy ΔF/F0
Distance to baseline @ 200 s
*
****
b(i)
b(ii)
b(iii)
Retigabine
c(i)
c(ii)
c(iii)
BMS191011
d(i)
d(ii)
d(iii)
Paxilline
**
e(i)
e(ii)
e(iii)
6 μM
XE-991

Supplementary Figure 6 – *Effect of potassium channel modulators on SV recycling.* Primary cultures of hippocampal neurons prepared from *Dnm1*$^{+/+}$ embryos were transfected with synaptophysin-pHluorin (sypHy) between 7-9 DIV. At 13-15 DIV, cultures were stimulated with a train of 300 action potentials (10 Hz) in the presence of 3 μM, 10 μM or 30 μM of either NS11021 (**a**), Retigabine (**b**) or BMS-911011 (**c**) or a vehicle control (DMSO). In identical separate experiments, cultures were stimulated in the presence of 10 μM Paxilline (**d**) or 6 μM XE-991 (**e**) or a vehicle control (DMSO). Cultures were pulsed with NH_4Cl imaging buffer 180 s after stimulation. (**all i**) Average sypHy response ($\Delta F/F_0$ ± SEM) to 10 Hz stimulation, normalised to stimulation (NS11021, n=6 DMSO, n=7 3 μM, n=8 10 μM, n=6 30 μM; Retigabine, n=10 DMSO, n=13 3 μM, n=14 10 μM, n=10 30 μM; BMS-911011, n=9 DMSO, n=10 3 μM, n=10 10 μM, n=12 30 μM; Paxilline, n=15 both DMSO and 10 μM; XE-991, n=13 both DMSO and 6 μM). (**all ii**) Average peak level of sypHy fluorescence ($\Delta F/F_0$ ± SEM) normalised to the NH_4Cl challenge (NS11021, one-way ANOVA, n=6 DMSO, n=7 3 μM, n=8 10 μM, n=6 30 μM, all ns; Retigabine, one-way ANOVA, n=10 DMSO, n=13 3 μM, n=14 10 μM, n=10 30 μM, all ns; BMS-911011, one-way ANOVA, n=9 DMSO, n=10 3 μM, n=10 10 μM, n=12 30 μM, all ns; Paxilline, Unpaired two-sided t test n=15 both DMSO and 10 μM, p=0.003; XE-991, Unpaired two-sided t test n=13 both DMSO and 6 μM, p=0.361). (**all, iii**) Average level of sypHy fluorescence ($\Delta F/F_0$ ± SEM) at 200 s (NS11021, one-way ANOVA, n=6 DMSO, n=7 3 μM, n=8 10 μM, n=6 30 μM, *p=0.011 DMSO vs 10 μM, ****p<0.00001 DMSO vs 30 μM; Retigabine, one-way ANOVA, n=10 DMSO, n=13 3 μM, n=14 10 μM, n=10 30 μM, all ns; BMS-911011, one-way ANOVA, n=9 DMSO, n=10 3 μM, n=10 10 μM, n=12 30 μM, all ns; Paxilline, Unpaired two-sided t test n=15 both DMSO and 10 μM, p=0.427; XE-991, two-sided Mann-Whitney test n=13 both DMSO and 6 μM, p=0.152). Source data are provided as a Source Data file.

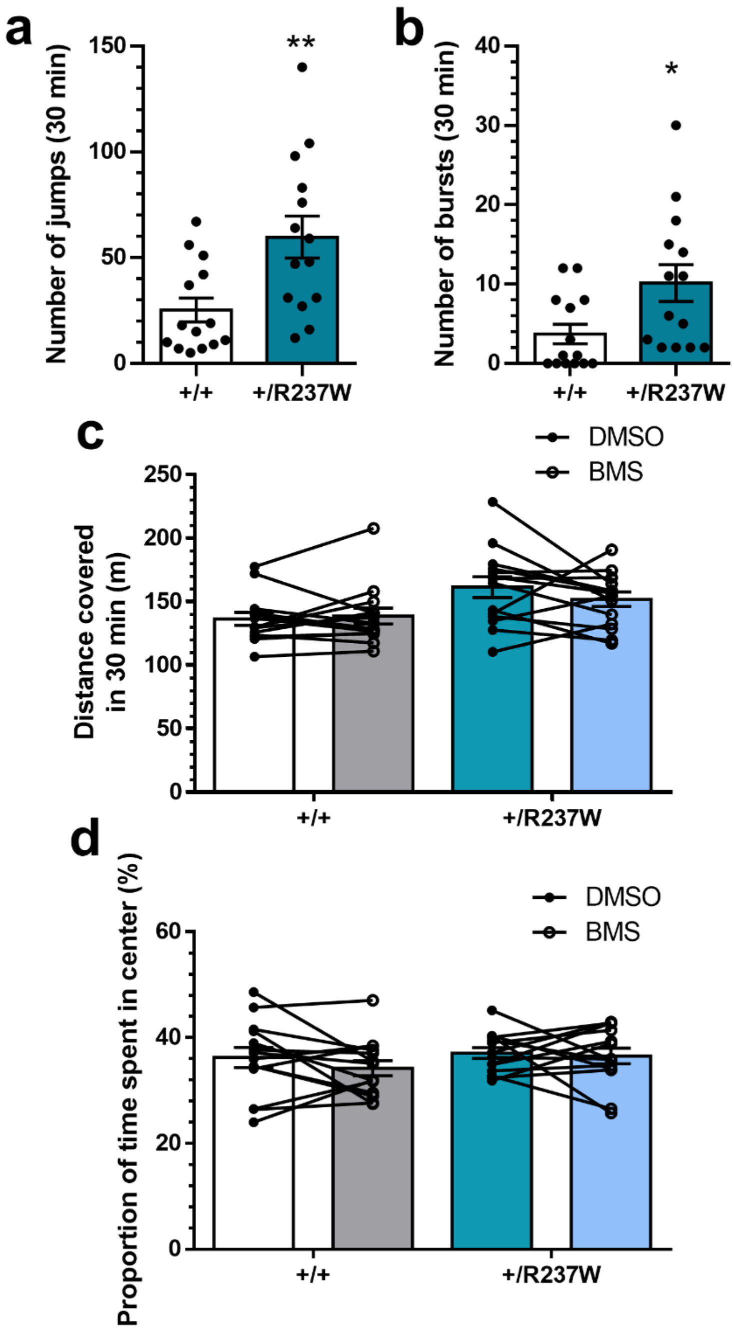

Supplementary Figure 7 – *Dnm1*$^{+/R237W}$ *mice display increased myoclonic jumping and jumping bursts in open field task.* *Dnm1*$^{+/+}$ and *Dnm1*$^{+/R237W}$ mice were placed in an open field chamber for a 30 min period for 5 days. After habituation on day 1, mice were dosed with either BMS-204352 or a vehicle control (DMSO) on days 2 and 4, and no treatment (washout) on days 3 and 5. Delivery of drug treatment was interleaved between days 2 and 4. (**a,b**) Comparison of *Dnm1*$^{+/+}$ and *Dnm1*$^{+/R237W}$ mice in the washout phase of day 5. Average number of myoclonic jumps (**a**), jumping bursts (**b**) is displayed ± SEM (Unpaired two-sided t test; n=14 for all; **a** ** p=0.0066; **b** * p=0.0238). (**c,d**) Comparison of *Dnm1*$^{+/+}$ and *Dnm1*$^{+/R237W}$ mice in the test phase (day 2 and 4). Average distance covered (**c**) and time spent in the centre of the area within the 30 min time period (**d**) is displayed ± SEM, General linear model (repeated measures) n=14 for all, all ns. Source data are provided as a Source Data file.

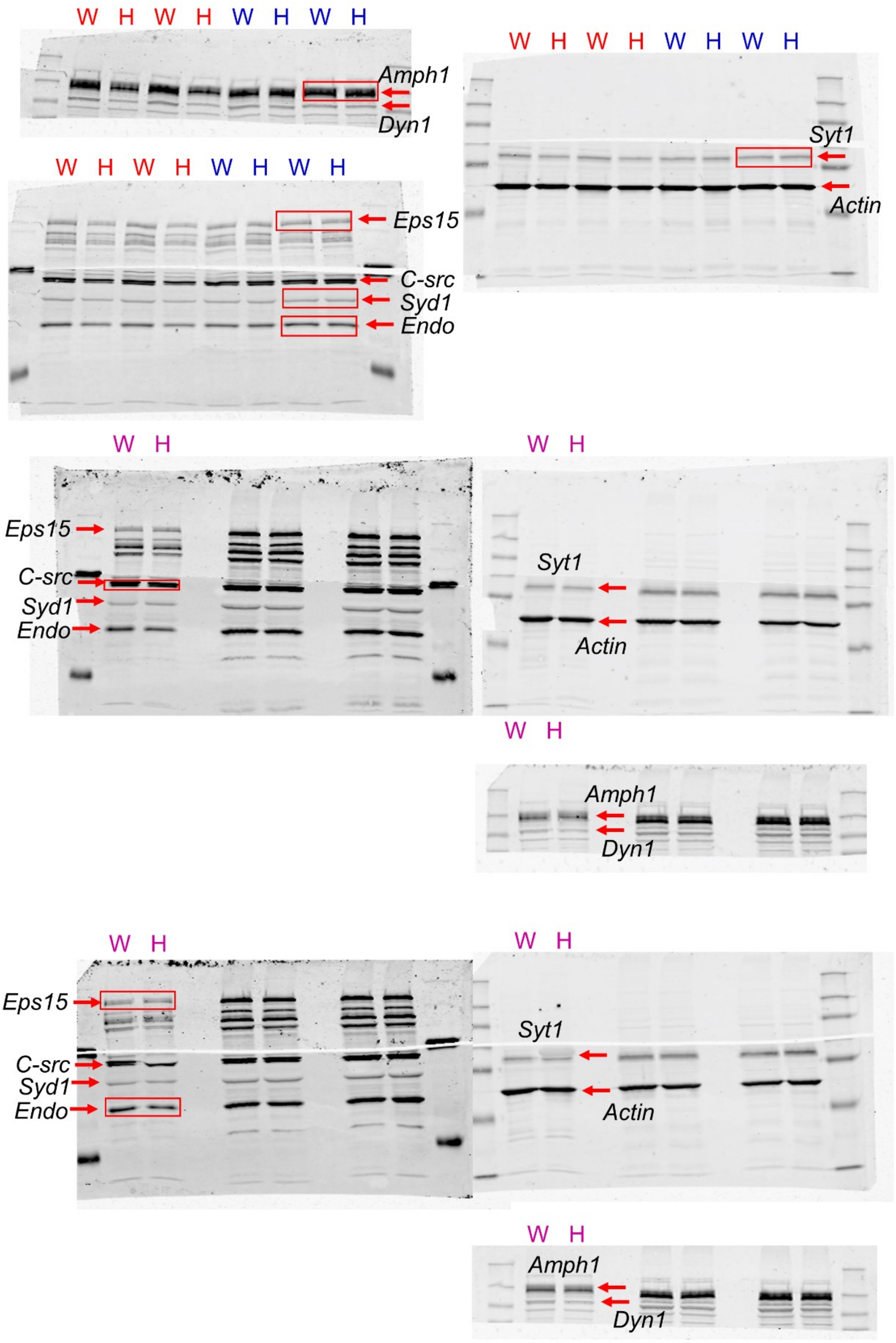

Uncropped blots for Figure 2b,c – Lysates from three independent primary cultures of hippocampal neurons were prepared from either *Dnm1*$^{+/+}$ (W) and *Dnm1*$^{+/R237W}$ (H) embryos blotted for Synaptotagmin-1 (Syt1), Amphiphysin-1 (Amph1), C-src, Endophilin (Endo), Syndapin (Syd1), Eps15 and Actin. Blots from the three independent experiments are displayed (red, blue and purple). Each sample was run twice as a technical replicate. Boxes indicate bands shown in Figure.

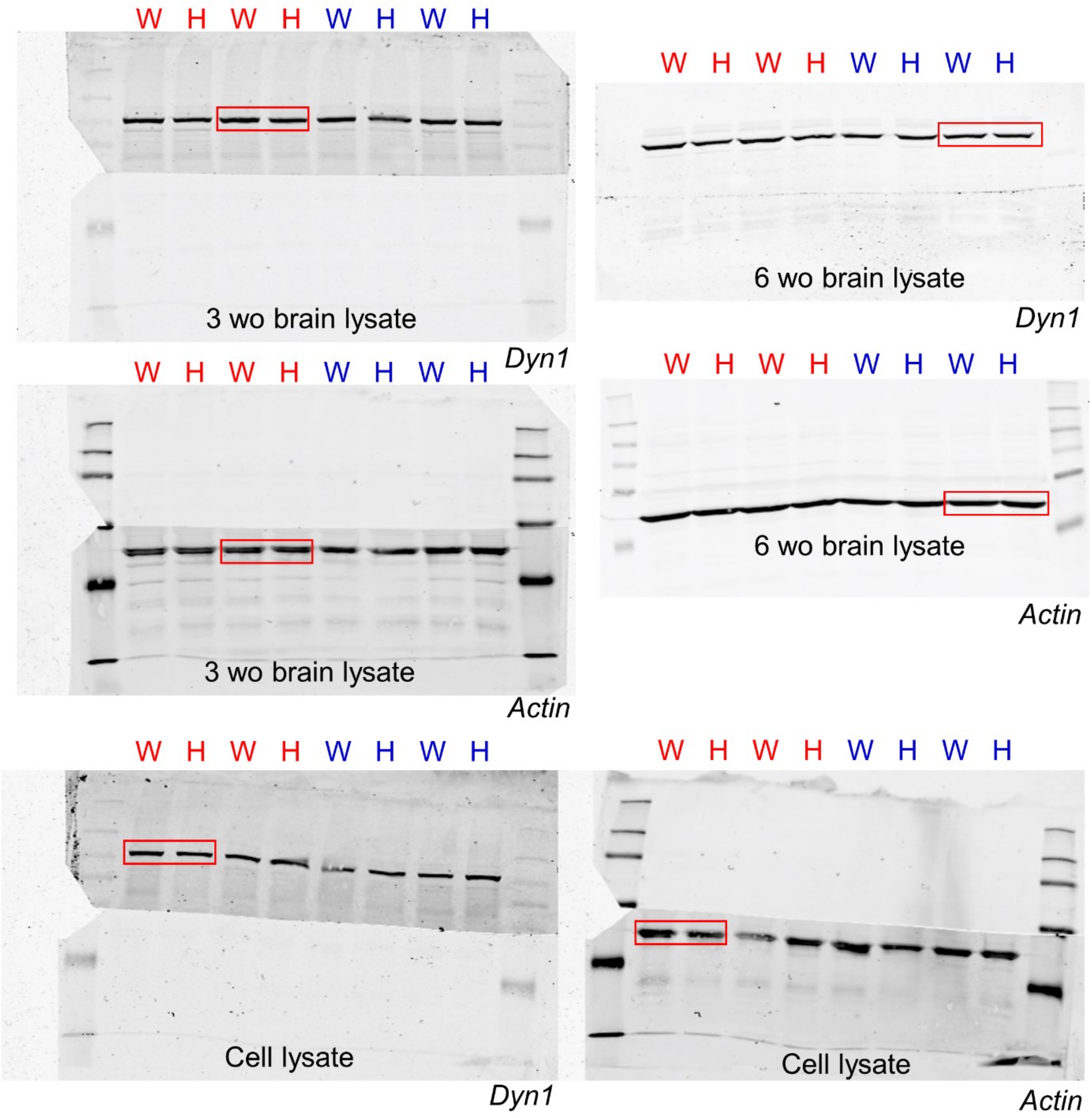

Uncropped blots for Figure S2a,b – Lysates from either primary cultures of *Dnm1*$^{+/+}$ (W) or *Dnm1*$^{+/R237W}$ (H) hippocampal neurons or from the brains of either 3 week or 6 week old *Dnm1*$^{+/+}$ (W) or *Dnm1*$^{+/R237W}$ (H) mice were prepared and blotted for dynamin-1 and actin. Blots from the two independent experiments are displayed (red, blue), with the other three indicated in uncropped blots from Figure 2b,c, Figure S2c,d and Figure S2e,f. Each sample was run twice as a technical replicate. Boxes indicate bands shown in Figure.

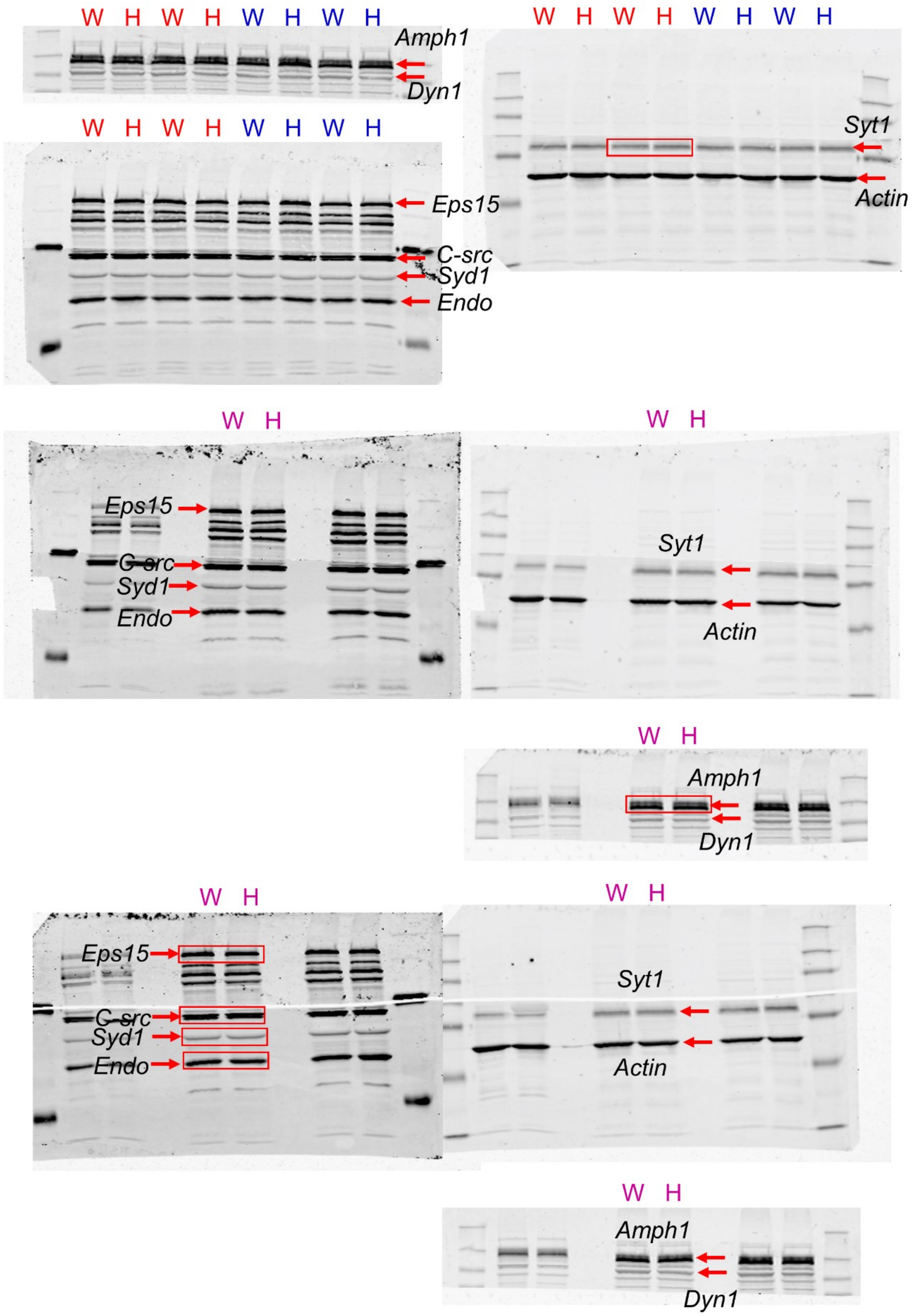

Uncropped blots for Figure 2Sc,d – Lysates from the brains of 3 week old *Dnm1*$^{+/+}$ (W) or *Dnm1*$^{+/R237W}$ (H) mice were prepared and blotted for Synaptotagmin-1 (Syt1), Amphiphysin-1 (Amph1), C-src, Endophilin (Endo), Syndapin (Syd1), Eps15 and Actin. Blots from the three independent experiments are displayed (red, blue and purple). Each sample was run twice as a technical replicate. Boxes indicate bands shown in Figure.

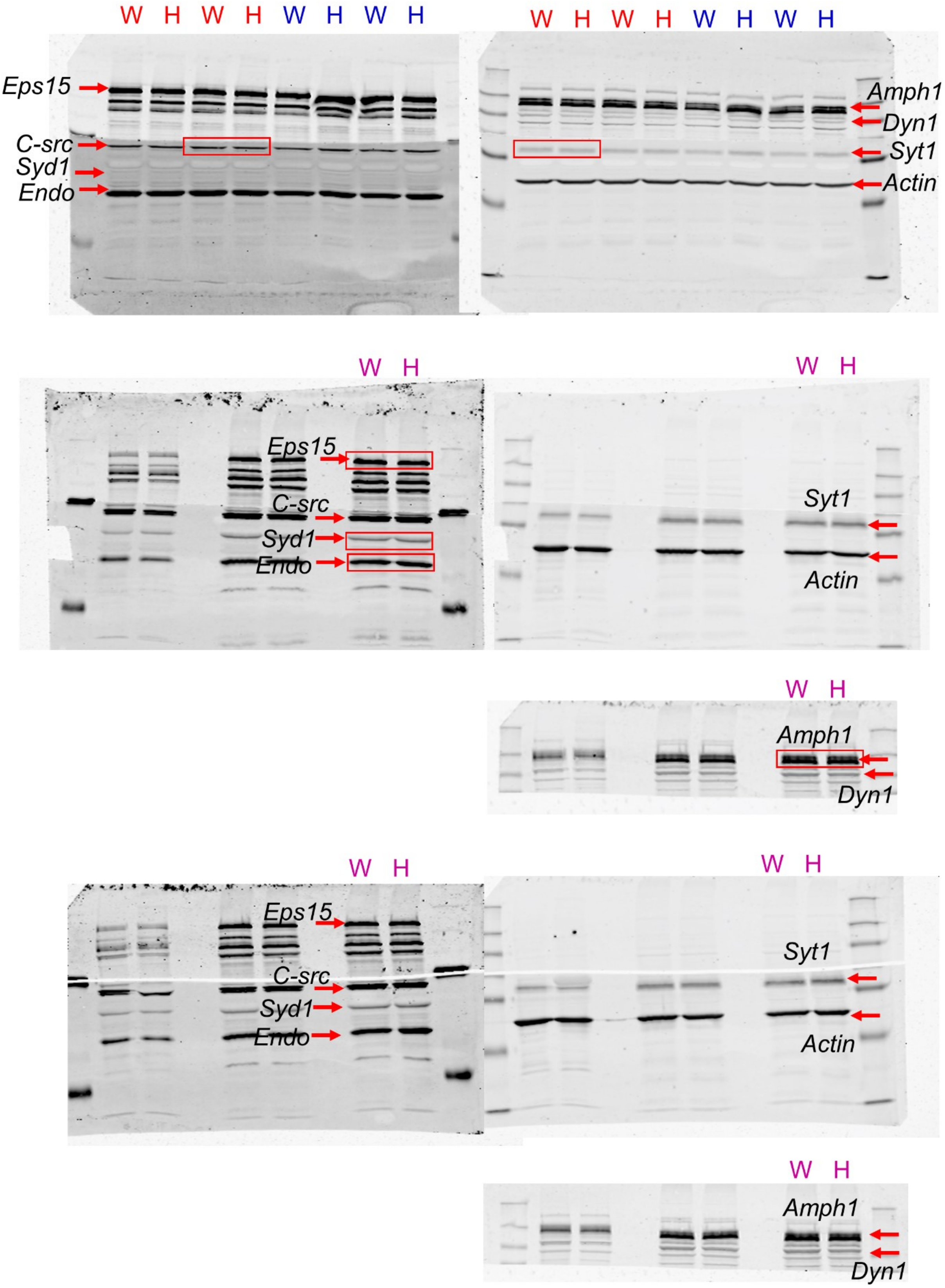

Uncropped blots for Figure 2Se,f – Lysates from the brains of 6 week old *Dnm1*$^{+/+}$ (W) or *Dnm1*$^{+/R237W}$ (H) mice were prepared and blotted for Synaptotagmin-1 (Syt1), Amphiphysin-1 (Amph1), C-src, Endophilin (Endo), Syndapin (Syd1), Eps15 and Actin. Blots from the three independent experiments are displayed (red, blue and purple). Each sample was run twice as a technical replicate. Boxes indicate bands shown in Figure.

	Standard aCSF	
	WT	Het
Resting membrane potential (mV)	-66.38	-65.20
Baseline (mV)	-70.76	-70.48
Step with 10 pA injection (mV)	-1.305	-1.332
Input resistance (MΩ)	130.5	133.2
Membrane decay time τ (ms)	18.26	21.17
Capacitance (MF)	139.3	160.8
Rheobase (pA)	107.9	103.9
AP threshold (mV)	-43.22	-44.47
AP peak (mV)	82.37	85.29
AP rise time 20 to 80% (ms)	0.1593	0.1765
AP width at half peak height (ms)	0.873	0.976
Max rise rate (mV/ms)	401.6	370.9
Max decay rate (mV/ms)	95.77	86.47
Rise/Decay	4.14	4.24
Sag current as % of steady state	24.9	28.7
Rebound potential (mV)	4.01	4.81
mAHP (mV)	-8.83	-8.35
fAHP (mV)	-4.78	-4.09
Max firing frequency (Hz)	50.7	49.4

Supplementary Table 1 – *Intrinsic properties of* $Dnm1^{+/R237W}$ *CA1 neurons*. Acute hippocampal slices from P19-25 $Dnm1^{+/+}$ or $Dnm1^{+/R237W}$ mice were prepared and the intrinsic properties of CA1 neurons were determined using whole-cell patch clamp recording. The parameters monitored are outlined above. Highlighted pairs = $p < 0.05$ unpaired two-tailed t test. Source data are provided as a Source Data file.

Figure 1	Group	Mean±SEM	n = # of experiments/coverslips N = # of neuronal preparations	Comparison	P	Statistical test
**Fig1a**	Empty	62.6 ± 1.1 μM	4	WT vs Empty	<0.0001	One-way ANOVA with Dunnett's multiple comparison test
	WT	119.6 ± 5.8 μM	4			
	K44A	93.6 ± 7.2 μM	4	WT vs K44A	0.0138	
**Fig1b**	Empty	63.8 ± 3.5 μM	3	WT vs Empty	<0.0001	
	WT	123.2 ± 2.7 μM	3			
	R237W	102.8 ± 3.9 μM	3	WT vs R237W	0.0093	
**Fig1d**	mCer	0.12 ± 0.034	9/4	WT vs mCer	0.978	
	WT	0.11 ± 0.039	10/4			
	K44A	0.31 ± 0.044	17/4	WT vs K44A	0.0046	
**Fig1e**	mCer	0.44 ± 0.036	9/4	WT vs mCer	0.086	
	WT	0.55 ± 0.044	10/4	WT vs K44A	0.472	
	K44A	0.50 ± 0.028	17/4			
**Fig1g**	WT	0.17 ± 0.047	16/5	WT vs R237W	0.0007	Unpaired t test
	R237W	0.50 ± 0.081	8/5			
**Fig1h**	WT	0.47 ± 0.042	16/5	WT vs R237W	0.480	
	R237W	0.43 ± 0.042	8/5			

Figure 2	Group	Mean±SEM	n = # of neuronal preparations	Comparison	P	Statistical test
**Fig2c**	WT Syt1	1.00 ± 0.053	3	WT vs R237W Syt1	0.900	Mann-Whitney test
	R237W Syt1	0.93 ± 0.12	3			
	WT Amph1	1.00 ± 0.076	3	WT vs R237W Amph1	0.700	
	R237W Amph1	0.91 ± 0.13	3			
	WT C-Src	1.00 ± 0.053	3	WT vs R237W C-Src	0.700	
	R237W C-Src	0.88 ± 0.17	3			
	WT Endo	1.00 ± 0.053	3	WT vs R237W Endo	0.400	
	R237W Endo	0.83 ± 0.12	3			
	WT Syd	1.00 ± 0.053	3	WT vs R237W Syd	0.900	
	R237W Syd	0.93 ± 0.12	3			
	WT Eps15	1.00 ± 0.053	3	WT vs R237W Eps15	0.900	
	R237W Eps15	0.90 ± 0.16	3			

Figure 3	Group	Mean±SEM	n = # of coverslips/ N = # of neuronal preparations	Comparison	P	Statistical test
**Fig 3b**	WT	0.106 ± 0.021	36 profiles	WT vs R237W	0.005	Mann-Whitney test
	R237W	0.255 ± 0.044	35 profiles			
**Fig 3c**	WT	10.15 ± 0.79	36 profiles	WT vs R237W	0.0441	
	R237W	11.91 ± 0.74	35 profiles			
**Fig 3d**	WT	945 ± 22.8	36 profiles	WT vs R237W	0.0024	Unpaired t test
	R237W	1061 ± 28.9	35 profiles			
**Fig 3g**	WT	0.12 ± 0.042	9/4	WT vs R237W	0.045	Mann-Whitney test
	R237W	0.29 ± 0.052	12/4			
**Fig 3h**	WT	0.33 ± 0.023	9/4	WT vs R237W	0.237	

	R237W	0.41 ± 0.031	12/4			Unpaired t test
Fig 3j	WT	0.13 ± 0.029	9/4	WT vs R237W	0.003	
	R237W	0.30 ± 0.037	11/4			
Fig 3k	WT	0.44 ± 0.0.38	9/4	WT vs R237W	0.751	
	R237W	0.42 ± 0.049	11/4			
Fig 3n	WT	3.34 ± 0.160	8/4	WT vs R237W	0.0393	
	R237W	2.90 ± 0.099	7/4			
Figure 4	Group	Mean±SEM	n = # slices / N = # of animals	Comparison	P	Statistical test
**Fig 4b**	WT		20/10	WT vs R237W	0.9971	Two-way ANOVA
	R237W		28/14			
**Fig 4c**	WT	107.9 ± 7.06	20/10	WT vs R237W	0.6129	Unpaired t test
	R237W	103.9 ± 4.44	28/14			
**Fig 4e**	WT	2.09 ± 0.28	10/6	WT vs R237W	0.0008	Mann-Whitney test
	R237W	0.92 ± 0.13	12/7			
**Fig 4f**	WT	-25.2 ± 2.32	10/6	WT vs R237W	0.665	
	R237W	-25.6 ± 1.50	12/7			
**Fig 4h**	WT	7.527 ± 0.946	9/5	WT vs R237W	0.3791	
	R237W	6.393 ± 0.460	12/6			
**Fig 4i**	WT	11.78 ± 1.40	9/5	WT vs R237W	0.0226	
	R237W	16.32 ± 0.873	12/6			
**Fig 4k**	WT		34/20	WT vs R237W Overall	0.0181	Two-way ANOVA
	R237W		39/23			
	25 µA WT	68.6 ± 7.33	34/20	WT vs R237W 25 µA	0.132	
	25 µA R237W	130.7 ± 18.12	39/23			
	50 µA WT	143.3 ± 18.50	34/20	WT vs R237W 50 µA	0.0428	
	50 µA R237W	237.7 ± 29.2	39/23			
	75 µA WT	204.1 ± 25.6	34/20	WT vs R237W 75 µA	0.0049	
	75 µA R237W	335.6 ± 37.3	39/23			
	100 µA WT	249.4 ± 30.5	34/20	WT vs R237W 100 µA	0.0004	
	100 µA R237W	499.0 ± 76.0	39/23			
**Fig 4m**	WT		36/20	WT vs R237W Overall	0.9266	
	R237W		40/27			
	25 µA WT	102.0 ± 9.6	36/20	WT vs R237W 25 µA	>0.9999	
	25 µA R237W	101.7 ± 8.7	40/27			
	50 µA WT	167.4 ± 9.8	36/20	WT vs R237W 50 µA	0.9916	
	50 µA R237W	157.8 ± 15.1	40/27			
	75 µA WT	231.8 ± 16.5	36/20	WT vs R237W 75 µA	0.9996	
	75 µA R237W	236.0 ± 21.3	40/27			
	100 µA WT	289.8 ± 18.1	36/20	WT vs R237W 100 µA	0.9728	
	100 µA R237W	302.9 ± 25.5	40/27			
**Figure 5**	Group	Mean±SEM	n = # slices / N = # of animals	Comparison	P	Statistical test

Fig 5b	WT		11/5	WT vs R237W Overall	<0.0001	Two-way ANOVA with Fisher's LSD
	R237W		10/6			
	10 ms WT	1.77 ± 0.092	11/5	WT vs R237W 10 ms	0.745	
	10 ms R237W	1.58 ± 0.09	10/6			
	20 ms WT	1.88 ± 0.15	11/5	WT vs R237W 20 ms	0.874	
	20 ms R237W	1.71 ± 0.096	10/6			
	50 ms WT	1.78 ± 0.10	11/5	WT vs R237W 50 ms	0.399	
	50 ms R237W	1.52 ± 0.082	10/6			
	100 ms WT	1.71 ± 0.11	11/5	WT vs R237W 100 ms	0.358	
	100 ms R237W	1.44 ± 0.099	10/6			
	200 ms WT	1.41 ± 0.16	11/5	WT vs R237W 200 ms	0.677	
	200 ms R237W	1.20 ± 0.024	10/6			
	500 ms WT	1.17 ± 0.087	11/5	WT vs R237W 500 ms	0.999	
	500 ms R237W	1.13 ± 0.069	10/6			
Fig 5d	WT		9/5	WT vs R237W Overall	0.0018	Two-way ANOVA
	R237W		13/7			
	10 ms WT	0.685 ± 0.035	9/5	WT vs R237W 10 ms	0.1845	
	10 ms R237W	0.959 ± 0.095	13/7			
	20 ms WT	0.785 ± 0.075	9/5	WT vs R237W 20 ms	0.6512	
	20 ms R237W	0.964 ± 0.141	13/7			
	50 ms WT	0.798 ± 0.074	9/5	WT vs R237W 50 ms	0.2035	
	50 ms R237W	1.076 ± 0.144	13/7			
	100 ms WT	0.810 ± 0.058	9/5	WT vs R237W 100 ms	0.6487	
	100 ms R237W	0.990 ± 0.069	13/7			
	200 ms WT	0.725 ± 0.410	9/5	WT vs R237W 200 ms	0.9652	
	200 ms R237W	0.826 ± 0.060	13/7			
	500 ms WT	0.831 ± 0.074	9/5	WT vs R237W 500 ms	>0.9999	
	500 ms R237W	0.824 ± 0.053	13/7			
Fig 5f	WT		12/6	WT vs R237W	0.0043	Two-way ANOVA
	R237W		13/6			
Fig 5g	WT		12/6	WT vs R237W	<0.0001	
	R237W		13/6			
Fig 5h	WT	0.0548 ± 0.058	12/6	WT vs R237W	0.0045	Mann-Whitney test
	R237W	0.0328 ± 0.0041	13/6			
Fig 5i	WT	44.3 ± 3.8	12/6	WT vs R237W	0.0229	
	R237W	33.0 ± 2.7	13/6			

Figure 7	Group	Mean±SEM	n = # coverslips/ N = # of neuronal preparations	Comparison	P	Statistical test
Fig 7b	DMSO	0.348 ± 0.06	14/5	DMSO vs 10 μM BMS	0.0581	One-way ANOVA with Dunnett's multiple comparison test
	10 μM BMS	0.145 ± 0.08	11/5			
	30 μM BMS	0.130 ± 0.05	10/5	DMSO vs 30 μM BMS	0.0467	
Fig 7c	DMSO	0.508 ± 0.04	14/5	DMSO vs 10 μM BMS	0.202	
	10 μM BMS	0.404 ± 0.04	11/5			

	30 μM BMS	0.400 ± 0.06	10/5	DMSO vs 30 μM BMS	0.186	
**Fig 7e**	WT DMSO	0.109 ± 0.036	19/5	WT DMSO vs WT BMS	0.9876	
	WT BMS	0.126 ± 0.044	15/5			
	R237W DMSO	0.276 ± 0.034	16/5	WT DMSO vs R237W DMSO	0.0183	
	R237W BMS	0.033 ± 0.037	15/5	WT DMSO vs R237W BMS	0.4952	
**Fig 7f**	WT DMSO	0.410 ± 0.024	19/5	WT DMSO vs WT BMS	0.538	
	WT BMS	0.366 ± 0.030	15/5	WT DMSO vs R237W DMSO	0.925	
	R237W DMSO	0.428 ± 0.029	16/5	WT DMSO vs R237W BMS	0.089	
	R237W BMS	0.326 ± 0.023	15/5			

Figure 8	Group	Mean±SEM	n = # of coverslips/ N = # of neuronal preparations<br>n = # slices / N = # of animals	Comparison	P	Statistical test
**Fig 8b**	WT DMSO			WT DMSO vs WT BMS	0.158	Two-way ANOVA with Sidaks multiple comparison test
	WT BMS					
	25 μA DMSO	95.4 ± 13.5	13/6	DMSO vs BMS 25 μA	>0.999	
	25 μA BMS	102.5 ± 13.5	11/7			
	50 μA DMSO	191.1 ± 35.5	13/6	DMSO vs BMS 50 μA	0.389	
	50 μA BMS	288.5 ± 28.4	11/7			
	75 μA DMSO	311.3 ± 48.9	13/6	DMSO vs BMS 75 μA	0.332	
	75 μA BMS	414.6 ± 44.1	11/7			
	100 μA DMSO	432.7 ± 68.2	13/6	DMSO vs BMS 100 μA	0.367	
	100 μA BMS	532.4 ± 54.8	11/7			
**Fig 8d**	R237W DMSO			R237W DMSO vs R237W BMS	0.0004	
	R237W BMS					
	25 μA DMSO	95.4 ± 13.5	13/6	DMSO vs BMS 25 μA	0.9546	
	25 μA BMS	102.5 ± 13.5	13/7			
	50 μA DMSO	191.1 ± 35.5	13/6	DMSO vs BMS 50 μA	0.7284	
	50 μA BMS	288.5 ± 28.4	13/7			
	75 μA DMSO	311.3 ± 48.9	13/6	DMSO vs BMS 75 μA	0.0410	
	75 μA BMS	414.6 ± 44.1	13/7			
	100 μA DMSO	432.7 ± 68.2	13/6	DMSO vs BMS 100 μA	0.0145	
	100 μA BMS	532.4 ± 54.8	13/7			
**Fig 8f**	WT DMSO		5/3	WT DMSO vs WT BMS	0.296	Two-way repeated measures ANOVA
	WT BMS		6/4	WT DMSO vs R237W DMSO	0.002	
	R237W DMSO		5/3	WT DMSO vs R237W BMS	0.939	
	R237W BMS		7/4	R237W DMSO vs R237W BMS	0.013	

Figure 9	Group	Mean±SEM	n = # animals	Comparison	P	Statistical test
**Fig 9b**	WT DMSO	11.7 ± 4.8	14	WT DMSO vs WT BMS	0.586	General linear model (repeated measures) with Bonferroni multiple comparison
	WT BMS	15.6 ± 4.1	14	WT DMSO vs R237W DMSO	0.021	
	R237W DMSO	40.2 ± 10.5	14	WT BMS vs R237W BMS	0.314	
	R237W BMS	22.4 ± 5.2	14	R237W DMSO vs R237W BMS	0.019	
**Fig 9c**	WT DMSO	0.71 ± 0.45	14	WT DMSO vs WT BMS	0.393	General linear model (repeated measures) with Bonferroni multiple comparison
	WT BMS	2.21 ±0.96	14	WT DMSO vs R237W DMSO	0.016	
	R237W DMSO	7.14 ± 2.45	14	WT BMS vs R237W BMS	0.874	
	R237W BMS	2.43 ± 0.93	14	R237W DMSO vs R237W BMS	0.011	

Figure S1	Group	Mean±SEM	n = # of experiments/coverslips/ N = # of neuronal preparations	Comparison	P	Statistical test
**Fig S1b**	Empty	1.00 ± 0.089	16/4	Empty vs WT	0.0015	One-way ANOVA with Dunnett's multiple comparison test
	WT	2.663 ± 0.402	10/4			
	K44A	2.397 ± 0.353	11/4	Empty vs K44A	0.0065	
	R237W	2.759 ± 0.536	9/4	Empty vs R237W	0.0012	
	A408T	2.147 ± 0.258	21/4	Empty vs A408T	0.0116	
**Fig S1c**	Empty	62.72 ± 1.26	3	Empty vs WT	<0.0001	
	WT	121.2 ± 5.24	3			
	A408T	122.5 ± 1.18	3	WT vs A408T	0.9873	
**Fig S1e**	WT	0.194 ± 0.046	16/5	Empty vs A408T	0.397	Unpaired t test
	A408T	0.260 ± 0.063	12/5			
**Fig S1f**	WT	0.475 ± 0.042	16/5	Empty vs A408T	0.948	
	A408T	0.478 ± 0.041	12/5			

Figure S2	Group	Mean±SEM	n = # of neuronal preparations or n = # of brains	Comparison	P	Statistical test
**Fig S2b**	WT Cell lysate	1.000 ± 0.132	5	WT vs R237W	0.801	Unpaired t test with Welch's correction
	R237W cell lysate	0.918 ± 0.282	5			
	WT 3 weeks	1.000 ± 0.067	3	WT vs R237W	0.359	
	R237W 3 weeks	0.870 ± 0.112	3			
	WT 6 weeks	1.000 ± 0.115	3	WT vs R237W	0.671	
	R237W 6 weeks	0.955 ± 0.099	3			
**Fig S2d**	WT Syt1	1.00 ± 0.051	3	WT vs R237W	0.400	

	R237W Syt1	1.076 ± 0.041	3			Mann-Whitney test
	WT Amph1	1.00 ± 0.008	3	WT vs R237W	0.200	
	R237W Amph1	1.111 ± 0.050	3			
	WT C-Src	1.00 ± 0.100	3	WT vs R237W	0.900	
	R237W C-Src	1.046 ± 0.057	3			
	WT Endo	1.00 ± 0.027	3	WT vs R237W	0.900	
	R237W Endo	1.044 ± 0.071	3			
	WT Syd	1.00 ± 0.040	3	WT vs R237W	0.700	
	R237W Syd	1.034 ± 0.037	3			
	WT Eps15	1.00 ± 0.035	3	WT vs R237W	0.900	
	R237W Eps15	1.029 ± 0.073	3			
**Fig S2f**	WT Syt1	1.00 ± 0.142	3	WT vs R237W	0.700	
	R237W Syt1	1.206 ± 0.222	3			
	WT Amph1	1.00 ± 0.068	3	WT vs R237W	0.900	
	R237W Amph1	1.103 ± 0.219	3			
	WT C-Src	1.00 ± 0.036	3	WT vs R237W	0.900	
	R237W C-Src	1.139 ± 0.203	3			
	WT Endo	1.00 ± 0.035	3	WT vs R237W	0.700	
	R237W Endo	1.184 ± 0.125	3			
	WT Syd	1.00 ± 0.002	3	WT vs R237W	0.700	
	R237W Syd	1.008 ± 0.095	3			
	WT Eps15	1.00 ± 0.030	3	WT vs R237W	0.900	
	R237W Eps15	1.057 ± 0.102	3			

Figure S4	Group	Mean±SEM	n = # of coverslips/ N = # of neuronal preparations	Comparison	P	Statistical test
**Fig S4a**	WT	4697 ± 364	23 profiles	WT vs R237W	0.892	Mann-Whitney test
	R237W	4472 ± 190	26 profiles			
**Fig S4c**	WT	0.352 ± 0.019	11/3	WT vs R237W	0.839	Unpaired t test
	R237W	0.345 ± 0.028	10/3			
**Fig S4e**	WT	0.340 ± 0.016	17/3	WT vs R237W	0.919	Mann-Whitney test
	R237W	0.345 ± 0.021	16/3			
**Fig S4f**	WT	23072 ± 1556	8/4	WT vs R237W	0.974	Unpaired t test
	R237W	23145 ± 1571	7/4			

Figure S5	Group	Mean±SEM	n = # of coverslips/ N = # of neuronal preparations	Comparison	P	Statistical test
**Fig S5a**	WT	1.08 ± 0.12	12/6	WT vs R237W	0.739	Mann-Whitney test
	R237W	1.02 ± 0.15	13/6			
**Fig S5b**	DMSO		10/3	DMSO vs 10 μM BMS	ns	Two-way ANOVA with Dunnett's multiple comparison test
	10 μM BMS		8/3			
	30 μM BMS		6/3	DMSO vs 30 μM BMS	<0.05 (100 s to 168 s)	
**Fig S5c**	DMSO	0.323 ± 0.06	10/3		0.424	

	10 μM BMS	0.234 ± 0.05	8/3	DMSO vs 10 μM BMS		One-way ANOVA with Dunnett's multiple comparison test
	30 μM BMS	0.152 ± 0.04	6/3	DMSO vs 30 μM BMS	0.092	
**Fig S5d**	DMSO	0.512 ± 0.04	10/3	DMSO vs 10 μM BMS	0.3894	
	10 μM BMS	0.424 ± 0.02	8/3			
	30 μM BMS	0.294 ± 0.05	6/3	DMSO vs 30 μM BMS	0.0217	

Figure S6	Group	Mean±SEM	n = # of coverslips/ N = # of neuronal preparations	Comparison	P	Statistical test
**Fig S6a(ii)**	DMSO	0.334 ± 0.037	6/2	DMSO vs 3 μM	0.606	One-way ANOVA with Dunnett's multiple comparison test
	3 μM NS11021	0.386 ± 0.0235	7/2			
	10 μM NS11021	0.401 ± 0.030	8/2	DMSO vs 10 μM	0.356	
	30 μM NS11021	0.412 ± 0.040	6/2	DMSO vs 30 μM	0.337	
**Fig S6a(iii)**	DMSO	0.041 ± 0.022	6/2	DMSO vs 3 μM	0.987	
	3 μM NS11021	0.058 ± 0.044	7/2			
	10 μM NS11021	0.238 ± 0.032	8/2	DMSO vs 10 μM	0.011	
	30 μM NS11021	0.689 ± 0.069	6/2	DMSO vs 30 μM	<0.0001	
**Fig S6b(ii)**	DMSO	0.393 ± 0.045	10/3	DMSO vs 3 μM	0.902	
	3 μM Retigabine	0.424 ± 0.038	13/3			
	10 μM Retigabine	0.428 ± 0.026	14/3	DMSO vs 10 μM	0.868	
	30 μM Retigabine	0.456 ± 0.051	10/3	DMSO vs 30 μM	0.597	
**Fig S6b(iii)**	DMSO	0.084 ± 0.046	10/3	DMSO vs 3 μM	>0.999	
	3 μM Retigabine	0.084 ± 0.038	13/3			
	10 μM Retigabine	0.072 ± 0.033	14/3	DMSO vs 10 μM	0.991	
	30 μM Retigabine	0.216 ± 0.031	10/3	DMSO vs 30 μM	0.054	
**Fig S6c(ii)**	DMSO	0.366 ± 0.037	9/4	DMSO vs 3 μM	0.471	
	3 μM BMS191011	0.306 ± 0.044	10/4			
	10 μM BMS191011	0.310 ± 0.031	10/4	DMSO vs 10 μM	0.513	
	30 μM BMS191011	0.276 ± 0.020	12/4	DMSO vs 30 μM	0.145	
**Fig S6c(iii)**	DMSO	0.074 ± 0.074	9/4	DMSO vs 3 μM	0.821	
	3 μM BMS191011	0.009 ± 0.056	10/4			
	10 μM BMS191011	0.026 ± 0.062	10/4	DMSO vs 10 μM	0.917	
	30 μM BMS191011	0.067 ± 0.054	12/4	DMSO vs 30 μM	0.999	
	DMSO	0.481 ± 0.030	15/3		0.003	

**Fig S6d(ii)**	10 μM Paxilline	0.376 ± 0.012	15/3	DMSO vs 10 μM		Unpaired t test
**Fig S6d(iii)**	DMSO	0.115 ± 0.038	15/3	DMSO vs 10 μM	0.427	
	10 μM Paxilline	0.072 ± 0.037	15/3			
**Fig S6e(ii)**	DMSO	0.456 ± 0.031	13/3	DMSO vs 6 μM	0.361	
	6 μM XE-991	0.406 ± 0.039	13/3			
**Fig S6e(iii)**	DMSO	0.123 ± 0.056	13/3	DMSO vs 6 μM	0.152	Mann-Whitney test
	6 μM XE-991	0.022 ± 0.047	13/3			

Figure S7	Group	Mean±SEM	n = # animals	Comparison	P	Statistical test
**Fig S7a**	WT	25.29 ± 5.61	14	WT vs R237W	0.0066	Unpaired t test with Welch's correction
	R237W	59.71 ± 9.92	14			
**Fig S7b**	WT	3.72 ± 1.25	14	WT vs R237W	0.0238	
	R237W	10.14 ± 2.31	14			
**Fig S7c**	WT DMSO	136.0 ± 5.1	14	N/A	0.198	General linear model (repeated measures)
	WT BMS	138.2 ± 6.3	14			
	R237W DMSO	161.1 ± 8.2	14			
	R237W BMS	151.4 ± 5.7	14			
**Fig S7d**	WT DMSO	36.2 ± 1.9	14	N/A	0.550	General linear model (repeated measures)
	WT BMS	34.2 ± 1.4	14			
	R237W DMSO	37.0 ± 1.0	14			
	R237W BMS	36.5 ± 1.5	14			

Supplementary Table 2 – *Collated table of experimental n, P values and statistical tests*. Source data are provided as a Source Data file.
