# Supplementary material for: Reversal of cell, circuit and seizure phenotypes in a mouse model of DNM1 epileptic encephalopathy
Source: Nat Commun. 2023 Aug 30;14:5285. doi: 10.1038/s41467-023-41035-w (PMC10468497; doi:10.1038/s41467-023-41035-w)
Supplement: Supplementary file 3 — Description of Additional Supplementary Files [file 41467_2023_41035_MOESM3_ESM.pdf]

Description of Additional Supplementary Files

Supplementary Data 1 – Table displaying proteins identified in synaptosomes derived from Dnm1+/+ and Dnm1+/R237W mice. Proteins significantly increased in Dnm1+/R237W synaptosomes are highlighted in orange (see column B for log2 ratio), whereas those decreased are highlighted in blue. Dynamin-1 is highlighted in green. Significance was determined using a two-sided Student's t-test, with p values corrected for multiple comparisons using the BenjaminiHochberg method.

Supplementary Data 2 – DAVID analysis of up and down-regulated proteins in Dnm1+/R237W synaptosomes.

Supplementary Movie 1 – Example of myoclonic jumping in the Dnm1+/R237W mouse. Movie displaying the myoclonic jumping phenotype. Myoclonic jumping can be observed at 18, 32, 35, 113,114, 119, 123, 124, 126, 129, 131, 132, 134, 135, 136, 147, and 175 s with bursts occurring between 113-114, 123-126 and 131-136 s.
