# Supplementary material for: Reversal of cell, circuit and seizure phenotypes in a mouse model of DNM1 epileptic encephalopathy
Source: Nat Commun. 2023 Aug 30;14:5285. doi: 10.1038/s41467-023-41035-w (PMC10468497; doi:10.1038/s41467-023-41035-w)
Supplement: Supplementary file 7 — Reporting Summary [file 41467_2023_41035_MOESM7_ESM.pdf]

nature portfolio

Corresponding author(s): Katherine Bonnycastle & Michael A. Cousin

Last updated by author(s): Aug 14, 2023

Reporting Summary

Nature Portfolio wishes to improve the reproducibility of the work that we publish. This form provides structure for consistency and transparency in reporting. For further information on Nature Portfolio policies, see our Editorial Policies and the Editorial Policy Checklist.

Statistics

For all statistical analyses, confirm that the following items are present in the figure legend, table legend, main text, or Methods section.

n/a	Confirmed	
☐	☒	The exact sample size (n) for each experimental group/condition, given as a discrete number and unit of measurement
☐	☒	A statement on whether measurements were taken from distinct samples or whether the same sample was measured repeatedly
☐	☒	The statistical test(s) used AND whether they are one- or two-sided<br>*Only common tests should be described solely by name; describe more complex techniques in the Methods section.*
☐	☒	A description of all covariates tested
☐	☒	A description of any assumptions or corrections, such as tests of normality and adjustment for multiple comparisons
☐	☒	A full description of the statistical parameters including central tendency (e.g. means) or other basic estimates (e.g. regression coefficient) AND variation (e.g. standard deviation) or associated estimates of uncertainty (e.g. confidence intervals)
☐	☒	For null hypothesis testing, the test statistic (e.g. F, t, r) with confidence intervals, effect sizes, degrees of freedom and P value noted<br>*Give P values as exact values whenever suitable.*
☒	☐	For Bayesian analysis, information on the choice of priors and Markov chain Monte Carlo settings
☒	☐	For hierarchical and complex designs, identification of the appropriate level for tests and full reporting of outcomes
☒	☐	Estimates of effect sizes (e.g. Cohen's d, Pearson's r), indicating how they were calculated

Our web collection on statistics for biologists contains articles on many of the points above.

Software and code

Policy information about availability of computer code

Data collection	LI-COR Image Studio Lite software (version 5.2) was used to collect Western blot analysis. Zen Pro 2012 software (Zeiss) was used to acquire fluorescence image data. Clampex 10 software (pClamp 10) was used for collection of electrophysiology data. Spectronaut version 15.0 was used to acquire mass spectrometry data. Open Ephys GUI was used for acquisition of in vivo LFP recordings.
Data analysis	GraphPad Prism 8.4.3 and IBM SPSS Statistics v29 were used for statistical analysis. The FIJI distribution of Image J (National Institutes of Health) was used to analyze images as described in the methods section. ShinyGO v0.76.2 and the STRING web tool (v 11.5) were used to analyze mass spectrometry data. The Stimfit software package or Clampfit (from the pClamp 10) were used for electrophysiology analysis. Local field potential analysis was performed using the MNE Python package (version 1.4.2). Jumping behaviour was scored using Behavioral Observation Research Interactive Software (BORIS v.7.9.24). DeepLabCut (v.2.1.10.4) was used to compare the movement and position of mice in behavioural tasks.

For manuscripts utilizing custom algorithms or software that are central to the research but not yet described in published literature, software must be made available to editors and reviewers. We strongly encourage code deposition in a community repository (e.g. GitHub). See the Nature Portfolio guidelines for submitting code & software for further information.

Data

Policy information about availability of data

All manuscripts must include a data availability statement. This statement should provide the following information, where applicable:

- Accession codes, unique identifiers, or web links for publicly available datasets
- A description of any restrictions on data availability
- For clinical datasets or third party data, please ensure that the statement adheres to our policy

Authors can confirm that all relevant data are included in the article and/or its supplementary information files.
The one exception is the proteomic data ,which is deposited on PRIDE. Project accession: PXD039667; Project name: Reversal of cell, circuit and seizure phenotypes in a mouse model of DNM1 epileptic encephalopathy; Project DOI: Not applicable; Project webpage: http://www.ebi.ac.uk/pride/archive/projects/PXD039667.
Databases used include - SynGO (https://www.syngoportal.org/), STRING web tool (https://string-db.org/) and Database for Annotation, Visualization and Integrated Discovery (DAVID, https://david.ncifcrf.gov/).

Human research participants

Policy information about studies involving human research participants and Sex and Gender in Research.

Reporting on sex and gender	No research involving human participants
Population characteristics	No research involving human participants
Recruitment	No research involving human participants
Ethics oversight	No research involving human participants

Note that full information on the approval of the study protocol must also be provided in the manuscript.

Field-specific reporting

Please select the one below that is the best fit for your research. If you are not sure, read the appropriate sections before making your selection.

☒ Life sciences ☐ Behavioural & social sciences ☐ Ecological, evolutionary & environmental sciences

For a reference copy of the document with all sections, see nature.com/documents/nr-reporting-summary-flat.pdf

Life sciences study design

All studies must disclose on these points even when the disclosure is negative.

Sample size	No statistical methods were used to predetermine sample sizes and no randomization procedures were applied. Sample sizes were estimated as a result of previous studies (Clayton et al (2010) Nature Neurosci 13, 845-851; Smillie et al (2013) Nature Commun 4, 2394; Nicholson-Fish et al (2015) Neuron 88, 973-984; Harper et al (2020) J Neurosci 40, 4586-4595; Ivanova et al. (2021) Sci Adv 7, eabf3873; Bonnycastle et al (2022) J Neurosci 42, 1618-1628) for all experiments except behavioral studies, where pilot experiments were performed.
Data exclusions	No data were excluded from the analysis.
Replication	All data within the study were found to be replicable in at least 3 independent experiments. The two exceptions are the gross anatomical study in Figure 2a, and the power analysis of local field potential recordings in Figure 6d,e (n=2 due to low number of wild-type mice that performed myoclonic jumping).
Randomization	No statistical methods were used to predetermine sample sizes and no randomization procedures were applied. Covariates were controlled by interleaving experimental conditions throughout the experimental procedure.
Blinding	Experimenters were blinded to the genotype of both animals and cells for all experiments and data analysis.

Reporting for specific materials, systems and methods

We require information from authors about some types of materials, experimental systems and methods used in many studies. Here, indicate whether each material, system or method listed is relevant to your study. If you are not sure if a list item applies to your research, read the appropriate section before selecting a response.

Materials & experimental systems

n/a	Involved in the study
☐	☒ Antibodies
☐	☒ Eukaryotic cell lines
☒	☐ Palaeontology and archaeology
☐	☒ Animals and other organisms
☒	☐ Clinical data
☒	☐ Dual use research of concern

Methods

n/a	Involved in the study
☒	☐ ChIP-seq
☒	☐ Flow cytometry
☒	☐ MRI-based neuroimaging

Antibodies

Antibodies used

Goat anti-amphyphysin-1 (Santa Cruz sc-8536)
Rabbit anti-Eps15 (Santa Cruz sc-534)
Goat anti-dynamin-1 (Santa Cruz sc-6402)
Mouse anti-synaptotagmin-1 (Abcam ab13259)
Rabbit anti-syndapin-1 (Abcam ab137390)
Goat anti-endophilin-A1 (Santa Cruz sc-10874)
Rabbit anti-C-src (Santa Cruz sc-19)
Mouse anti-actin (Sigma Aldrich A4325)
Mouse anti-NeuN (Merck MAB377)
Donkey anti-goat (red, Li-Cor, 734 926-68074)
Donkey anti-goat (green, Li-Cor, 926-32214)
Donkey anti-rabbit (green, Li-Cor, 926-32213)
Donkey anti-mouse (green, Li-Cor, 926-32212)
Goat anti-mouse (red, Li-Cor, 926-68070)
Donkey anti-rabbit Alexa Fluor 568 (Invitrogen A10042)
Chicken anti-GFP (Abcam ab13970)
Rabbit anti-SV2A (Abcam 32942)
Goat anti-dynamin-1 (Santa Cruz sc-6402)
Goat anti-chicken Alexa-Fluor-488 (Invitrogen A11039)
Goat anti-rabbit Alexa-Fluor-568 (Invitrogen A21069)
Donkey anti-goat Alexa-Fluor-647 (Invitrogen A21447)

Validation

Anti-amphyphysin-1, Western blotting, see manufacturer's references
Anti-Eps15, Western blotting, see manufacturer's references
Anti-dynamin-1, Western blotting, see manufacturer's references
Anti-synaptotagmin-1, Western blotting, see manufacturer's references
Anti-syndapin-1, Western blotting, see manufacturer's references
Anti-endophilin-A1, Western blotting, see manufacturer's references
Anti-C-src, Western blotting, see manufacturer's references
Anti-actin, Western blotting, see manufacturer's references
Donkey anti-goat (red, Li-Cor, 734 926-68074), Western blotting, see manufacturer's references
Donkey anti-goat (green, Li-Cor, 926-32214), Western blotting, see manufacturer's references
Donkey anti-rabbit (green, Li-Cor, 926-32213), Western blotting, see manufacturer's references
Donkey anti-mouse (green, Li-Cor, 926-32212), Western blotting, see manufacturer's references
Goat anti-mouse (red, Li-Cor, 926-68070), Western blotting, see manufacturer's references
Donkey anti-rabbit Alexa Fluor 568 (Invitrogen A10042), Immunocytochemistry, see manufacturer's references
Anti-GFP, Immunohistochemistry, see manufacturer's references
anti-SV2A, Immunohistochemistry, see manufacturer's references
Anti-dynamin-1, Immunohistochemistry, see manufacturer's references
Anti-chicken Alexa-Fluor-488, Immunohistochemistry, see manufacturer's references
Anti-NeuN, Immunocytochemistry, see manufacturer's references
Goat anti-rabbit Alexa-Fluor-568, Immunohistochemistry, see manufacturer's references
Donkey anti-goat Alexa-Fluor-647, Immunohistochemistry, see manufacturer's references
Donkey anti-goat Alexa-Fluor-647 (Invitrogen A21447)

Eukaryotic cell lines

Policy information about cell lines and Sex and Gender in Research

Cell line source(s)

HEK293T cells were originally obtained from ATCC

Authentication

HEK293T cells were commercially purchased. The manufacturer tested and authenticated these cells. No other authentication procedure was performed on these cells, since they were used purely to produce protein, and not for functional assays.

Mycoplasma contamination

All cell lines were periodically tested for mycoplasma and all tested negative.

Commonly misidentified lines (See ICLAC register)	Not used in this study

Animals and other research organisms

Policy information about studies involving animals; ARRIVE guidelines recommended for reporting animal research, and Sex and Gender in Research

Laboratory animals	Wild-type C57Bl/6J mice (Dnm1+/+) were maintained in an in-house colony (original source, Charles River) and were used as a source of tissue for wild-type hippocampal cultures in experiments where dynamin-1 variants were overexpressed. The Dnm1+/R237W mouse was generated by Horizon Discovery (St. Louis, USA). Briefly, the codon encoding R237 within the Dnm1 gene was targeted using CRISPR-Cas9 technologies on a C57Bl/6J genetic background. This resulted in the modification of the Dnm1 gene sequence from CGGAGC (equivalent amino acids 237/238 - RS) to TGGTCT (amino acids 237/238 - WS). Embryonic tissue was used for primary neuronal culture. Animals either 3 weeks or 6 weeks old were used for preparation of whole brain lysates. Brain slices for immunohistochemistry and synaptosome preparation was performed on 2-month old animals. Electrophysiology was performed on brain slices from mice aged P19-25. In vivo local field potential recordings were performed on 8-week old mice. Behavioural studies were performed on mice aged 6-8 weeks.
Wild animals	This study did not involve wild animals.
Reporting on sex	The experiments did not differentiate between the sex of the animals, with the exception of immunohistochemistry studies and production of synaptosomes for mass spectrometry analysis.
Field-collected samples	The study did not involve samples collected from the field.
Ethics oversight	Animal work was performed in accordance with the UK Animal (Scientific Procedures) Act 1986, under Project and Personal Licence authority and was approved by the Animal Welfare and Ethical Review Body at the University of Edinburgh (Home Office project licences – 7008878 and PP5745138 to Prof. Cousin and PP1538548 to Dr. Gonzalez-Sulser).

Note that full information on the approval of the study protocol must also be provided in the manuscript.
